# Taking a look at your speech: identifying diagnostic status and negative symptoms of psychosis using convolutional neural networks

**DOI:** 10.1038/s44277-025-00040-1

**Published:** 2025-07-08

**Authors:** Gleb Melshin, Anthony DiMaggio, Nadia Zeramdini, Michael MacKinley, Lena Palaniyappan, Alban Voppel

**Affiliations:** 1 Douglas Research Centre, McGill University, Montreal, QC Canada; 2 Faculty of Medicine, McGill University, Montréal, QC Canada; 3 University of Toronto, Toronto, ON Canada; 4 Lawson Health Research Institute, University of Western Ontario, London, ON Canada; 5 Department of Psychiatry, McGill University, Montreal, QC Canada; 6 Robarts Research Institute, University of Western Ontario, London, ON Canada

**Keywords:** Diagnostic markers, Schizophrenia

## Abstract

Speech-based indices are promising objective biomarkers for identifying schizophrenia and monitoring symptom burden. Static acoustic features show potential but often overlook time-varying acoustic cues that clinicians naturally evaluate—such as negative symptoms—during clinical interviews. A similar dynamic, unfiltered approach can be applied using speech spectrograms, preserving acoustic-temporal nuances. Here, we investigate if this method has the potential to assist in the determination of diagnostic and symptom severity status. Speech recordings from 319 participants (227 with schizophrenia spectrum disorders, 92 healthy controls) were segmented into 10 s fragments of uninterrupted audio (*n* = 110,246) and transformed into log-Mel spectrograms to preserve both acoustic and temporal features. Participants were partitioned into training (70%), validation (15%), and test (15%) datasets without overlap. Modified ResNet-18 convolutional neural networks (CNNs) performed three classification tasks; (1) schizophrenia-spectrum vs healthy controls, within 179 clinically-rated patients, (2) individuals with more severe vs less severe negative symptom burden, and (3) clinically obvious vs subtle blunted affect. Grad-CAM was used to visualize salient regions of the spectrograms that contributed to classification. CNNs distinguished schizophrenia-spectrum participants from healthy controls with 87.8% accuracy (AUC = 0.86). The classifier trained on negative symptom burden performed with somewhat less accuracy (80.5%; AUC = 0.73) but the model detecting blunted affect above a predefined clinical threshold achieved 87.8% accuracy (AUC = 0.79). Importantly, acoustic information contributing to diagnostic classification was distinct from those identifying blunted affect. Grad-CAM visualization indicated that the CNN targeted regions consistent with human speech signals at the utterance level, highlighting clinically relevant vocal patterns. Our results suggest that spectrogram-based CNN analyses of short conversational segments can robustly detect both schizophrenia-spectrum disorders and ascertain burden of negative symptoms. This interpretable framework underscores how time–frequency feature maps of natural speech may facilitate more nuanced tracking and detection of negative symptoms in schizophrenia.

## Lay summary

Short snippets of everyday speech hold medical clues. Using computer vision techniques on sound pictures (“spectrograms”) from short speech fragments, researchers trained a neural network on recordings from 227 people with schizophrenia and 92 without. The model spotted schizophrenia with 88 % accuracy and judged negative symptom severity well, especially the “blunted affect” symptom. Visualizations showed it focused on speech patterns, not noise. The findings suggest analyzing speech transformed to pictures could help diagnose and track schizophrenia.

## Introduction

Speech disturbances constitute a defining feature of schizophrenia spectrum disorders (SSD) [1, 2]. These are immediately apparent when considering a set of symptoms referred to as negative symptoms - alogia, poverty of speech, and diminished spontaneous conversation - that profoundly impede social and functional outcomes [3, 4]. Clinically, these behaviors are evaluated largely through direct observation of patients’ speech patterns and conversational engagement [5]. In this sense, these features are more of observed ‘signs’ than reported ‘symptoms’ as individuals do not actively complain about them and struggle to provide specific verbal descriptions. In particular, features such as blunted affect carry diagnostic significance and prognostic value, but are not assessed reliably in clinical practice [6, 7]. Clinical assessment of these features can greatly benefit from the availability of complementary objective markers that are readily accessible.

Recent studies suggest that carefully selected and engineered acoustic features—including derivatives of pitch, prosody, pauses, and overall speaking rate—can serve as potential markers for identifying SSD and capturing its symptom severity [8–10]. These studies, based on readily accessible software packages have advanced the replicability of speech-based analyses [11–13]. These programs have allowed a large number of features to be extracted from very short time windows - as short as 50 ms, risking redundancy and overfitting. In turn, researchers have resorted to data summarisation or guided selection before further compressing the feature space to reduce dimensionality ([9, 14, 15] for examples). This workflow requires some presupposition of feature relevance and theories that are not often explicit (i.e., expert knowledge) to mitigate complexity. Such a feature engineering process is thought by some to be a major bottleneck for generalisability and clinical implementation for machine learning applications [16]. Our goal is to exploit temporal acoustic information from short segments of speech without any selection, manipulation and transformation of raw data (i.e., no feature engineering) to test if ‘acoustic separation’ of schizophrenia is feasible on the basis of speech spectra. To this end, we employ a deep-learning convolutional neural network (CNN) shown to be successful in developing models to detect depression, bipolar disorder and sleep disorders [17–19].

One of the major drawbacks in summarising acoustic information to a handful of features is the loss of temporal information. The moment-to-moment shifts in pitch, speaking rate, or pause structure serve as clinically informative cues to negative symptoms. Subtle changes in intonation over the course of an utterance, variations in speech rate tied to conversational dynamics, or specific junctures where pauses reveal heightened cognitive load [20] are all critical cues to assess one’s affect. Capturing these finer-grained temporal patterns is especially important for schizophrenia, where aberrant communication can manifest as inconsistent speech rhythms such as long delays before responses [21], pressured speech [22], or truncated sentence structures including blocking [23]. In this work, we exploit the raw temporal trace of speech, addressing the open question of the importance of subtle acoustic fluctuations in identifying the mental states that define schizophrenia and its severity.

Convolutional neural networks (CNNs), a method in the field of deep learning, offer a powerful solution to learn hierarchical representations directly from the temporal acoustic data available in speech spectrogram “images,” which has previously been applied to speech in schizophrenia [24]. Other deep learning approaches such as wav2vec [25, 26] exist and have been used in schizophrenia [27–30], we focus here on CNN spectrograms because they preserve both subtle acoustic cues, such as formant transitions, and the short-term temporal variations—like prolonged pauses—that might indicate clinical phenomena. Trained CNN models are highly scalable, handling large datasets efficiently and incorporating new data with minimal manual preprocessing [24, 31].

Building on this design, we deploy a CNN-based model to classify short, 10 s speech segments in three distinct tasks: (1) discriminating SSD from healthy controls, (2) dividing individuals into higher vs lower severity of overall negative symptoms, and (3) detecting blunted affect scores above a clinical threshold. By focusing on brief audio segments, we aim to preserve time-sensitive acoustic variation while keeping computational demands manageable. Our goal is not highest-in-field performance, but to show the feasibility of using CNNs in an heterogeneous sample using separated datasets. Recent systematic reviews have shown that clinical prediction models in psychiatry report high classification accuracy (area under the curve or AUC of 0.70–0.85), but >90% of prior studies were at high risk of bias due to overfitting or lack of robust out-of-sample validation [32]. In psychosis, reported AUCs of successful models are around 0.75–0.85 [33]. On this basis, we hypothesize that integrated acoustic and temporal features in a CNN will achieve clinically meaningful out-of-sample classification accuracy (AUC > 0.75) for diagnosis and to identify negative symptom severity in order to show the feasibility of a CNN-based approach. We apply Gradient-weighted Class Activation Mapping (Grad-CAM), a visualisation approach, to verify that the neural network’s attention converges on clinically interpretable parts of the spectrogram rather than on incidental noise.

## Patients and methods

### Participants

Data from 227 individuals with psychosis at various illness stages, (at-risk stage, medication-naive first episode psychosis, to chronic illness lasting >10 years) and 92 age-matched healthy controls from London, Ontario or Montreal, Canada were included. This sample overlaps partially with 2 prior studies [34, 35]. All participants gave informed consent to have their speech recorded and analysed. Recordings were in English, the preferred language of daily communication for all included participants. Speech was collected through recording the entire DISCOURSE protocol (www.discourseinpsychosis.org), which combines both open-ended and structured tasks to generate a range of speech styles. These tasks include free conversation, personal and health narratives, and picture-based descriptions, aimed at a duration of 20 min. For most participants, the full protocol was conducted, capturing spontaneous, semi-structured and structured speech. However, in the case of untreated, acutely unwell individuals, only the short picture-description segment was administered—consisting of three Thematic Apperception Test images, each described for 1 min [36] for a total duration of 3 min. Patients were diagnosed using DSM-5 operational criteria through a best-estimate consensus procedure (treating psychiatrist and the clinical research team) based on all available clinical information [37]. When patients were recruited from a first-episode psychosis clinic, the individual diagnoses were confirmed after 6–12 months to ensure diagnostic stability. Individual diagnostic distributions are shown in Table 1. Given the diagnostic heterogeneity, we use the term SSD here to capture all patients as they satisfied DSM-5 criterion A of schizophrenia or CHR criteria for schizophrenia (as described in [35]) at the initial presentation. Only 2 of the 17 clinical high risk subjects developed first episode psychosis (schizophrenia) in the subsequent 12 months, but they were included along with other SSD groups as the speech samples were obtained prior to the diagnostic outcome. All participants gave informed consent, and the Research Ethics Board at Western University approved the study.

**Table 1:** Demographic characteristics of SSD patients against HCs.

Category		SSD patients (*n* = 227)	Healthy controls (*n* = 91)	Statistics
Age				
Years	M (SD)	27.16 (8.94)	25.44 (5.85)	*F* = *2.829, p* = *0.0936*
Sex				
Male	*n* (%)	175 (77.09)	58 (63.74)	*X²* = *5.5661, p* = *0.018*^a^
Clinical scores				
PANSS-8	M	20.96 (8.00)	8.83 (0.00)	*F* = *176.4, p* < *0.001*^b^
N1, N4, N6	M (SD)	7.85 (4.70)	3 (0.00)	*F* = *87.17, p* < *0.001*^b^
Diagnosis				
Schizophrenia	*n* (%)	96 (42)		
Psychosis NOS	*n* (%)	18 (8)		
FEP	*n* (%)	72 (32)		
Schizoaffective	*n* (%)	20 (9)		
Bipolar with psychotic features	*n* (%)	4 (2)		
CHR	*n* (%)	17 (7)		

*M* mean, *SD* standard deviation, *PANSS* positive and negative syndrome scale, *NOS* not otherwise specified, *FEP* first episode psychosis, *CHR* clinical high risk.

^a^Indicates *p* value < 0.02.

^b^Indicates *p* value < 0.001.

Clinical symptoms were assessed in the same week as speech recordings for 179 patients and 82 controls using at least 8 items [38] of the PANSS scale [39] by trained RAs supervised by the same clinician (LP) for every case. All RAs achieved a minimum ICC of 0.85 for the PANSS total scores with LP at the end of their training (6 subjects or more, fixed raters, single measures), and item level discrepancies during the data acquisition were resolved by substituting uncertain scores with LP’s scores for all cohorts.

For symptom severity classification, only the 179 patients whose PANSS scores were available were included. To assess whether our approach is sensitive to overall negative burden and allowing measurement of symptom specificity, we first perform classification on average negative symptom levels, before focusing on the specific symptom of blunted affect. While acoustics are intuitively linked to blunted affect as a clinical sign, given the importance of determining overall negative symptom severity as being above or below a threshold that prompts clinical actions we use the median split that provides balanced sample size for this dataset. The negative PANSS scores were computed by taking the sum of N1 (blunted affect), N4 (passive/apathetic social withdrawal) and N6 (lack of spontaneity and flow of conversation) (possible range of 1-absent to 7-extreme for each). We performed a median split on the PANSS-8 negative symptom N1+N4+N6 items average. This median split approach (per item average of the cut-off = 2.62; median across 3 items = 7.85) formed one sub-group satisfying low negative symptoms (i.e., ≤3 average item score), and the other with higher burden that is likely to be functionally intrusive as per PANSS item descriptions, the cutoff 3 being the commonly used symptom remission criteria that denotes a feature being absent, minimal or mild [40]. To detect blunted affect, we divided patients into two groups having a PANSS N1 (blunted affect) of either ≤3 or >3. Sex differences were assessed with a chi-square test; age and symptom severity with ANOVAs. Statistical tests were performed in R [41].

### Creation of spectrograms

**Fig. 1:**
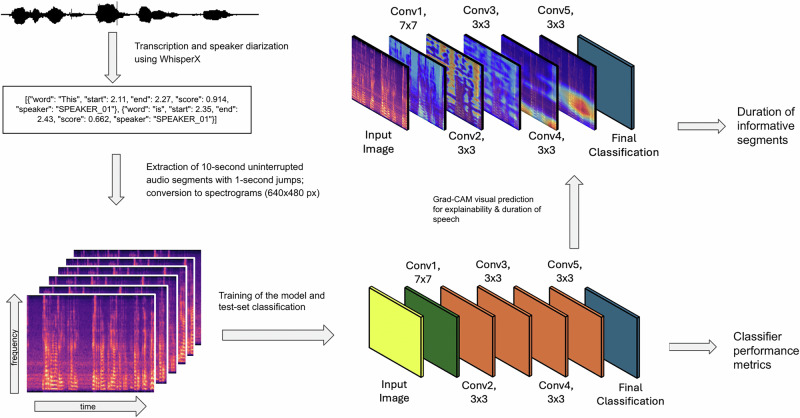
Flowchart of the general processing pipeline, going from the raw audio signal (top left) to 10 s spectrograms based on time stamped transcription (bottom left), binary classification through a resnet-18 based convolutional neural network into either patient-control, median negative symptom or clinical blunted affect classifiers (bottom right), with accompanying grad-CAM visualization of convolutional layers, showing log-mel spectrograms areas of interest (top right).

See Fig. 1 for an overview of the processing flow. Each interview audio file was transcribed using an offline implementation of WhisperX, an adaptation of OpenAI’s Whisper automatic speech recognition (ASR) system [42]. The output consisted of the transcribed text, per-word start and end timestamps, as well as the confidence score and the speaker associated with each word. Then, a list of fragments of interest of the patient’s speech was generated. The list of fragments of interest contained the timestamps of 10 s long, uninterrupted audio fragments. The cutoff of 10 s removes occurrences of crosstalk or short answers, where speaker identification is harder to estimate. A subset of audio fragments was manually checked for accuracy of the pipeline. All audio was re-sampled to 16,000 Hz and a log Mel spectrogram for each fragment of interest was created with fast Fourier transforms computed on windows of a length of 128 ms and a stride of 16 ms. All audio manipulations were done using librosa, a Python library for audio analysis [43].

### Training of convolutional neural network

Fully independent training, validation, and test sets are crucial for obtaining reliable performance estimates in real-world settings [44–46]. Yet in psychiatric research, large and heterogeneous datasets can be difficult to obtain, leading many to employ cross-validation or data augmentation [32, 33, 47]. Data augmentation and cross-validation can help offset data scarcity; these solutions need careful implementation to reduce additional noise and overfitting. Furthermore, when multiple speech segments from the same participant appear in both training and validation folds, cross-validation can inadvertently leak information and overestimate performance. In the present study, we minimize these concerns by enforcing a strict train–test–validation split at the participant level, thereby preserving ecological validity in our assessments. 70% of the patients were part of the training data set, while 15% of patients were part of the testing and 15% of the validation data sets. The proportions of spectrograms from each study in the original data set were respected in train, validation and testing sets.

ResNet-18 with a modified final fully-connected (FC) layer to accommodate for the two classes (see Fig. 1) was used in order to accomplish the training. In order to optimize the training and to achieve the best accuracy, we conducted empirical evaluations by testing a range of different parameters. The optimal hyperparameters which provided the best performance for our model were determined to be a batch size of 32, an initial learning rate of 0.001, 50 epochs, and a weight decay of 0.01. Hyperparameters were finalised before any evaluation on the held-out test cohort, and we did not run cross-validation, opting for a single stratified split that reflects intended clinical deployment while preventing participant-level leakage and preserving heterogeneity across folds. Classification metrics for binary classification such as Area Under the Curve (AUC), F1 scores, accuracy, precision and recall were collected for the SSD vs HC, negative symptom and blunted affect detection classifiers.

In order to compare our approach with other methodologies, we train two other models on the same train/validation/test split. First, the 88 eGeMAPSv02 standard features were extracted using OpenSMILE 13 and classified with a random forest. Second, we fine-tuned a wav2vec2 model [25, 48] for the three tasks as a reference; see Supplementary Table S2 for additional details.

To evaluate specificity, we tested the trained blunt affect symptom detection model on the overall severity-based classification task division, thereby checking if general illness severity acted as a confounding factor. We anticipated poorer performance on the severity split if the models were genuinely capturing patterns unique to the symptom of blunted affect.

### Feature visualization

Gradient-weighted Class Activation Mapping (Grad-CAM) [49] was used in order to provide visual insights into both the learning process and the final classification. This method generates a heatmap highlighting the regions of an image that most impact its classification by a CNN. It does so by calculating averaged gradients across feature maps and turning them into a heatmap which is then placed over the image. To determine whether the network captured short phonemic segments or entire phrases, we set a threshold at 63% of the maximum activation and measured the largest continuous horizontal extent above that cutoff. Grad-CAM activations of short durations would suggest reliance on a single word or a few phonemes, whereas longer spans would indicate that a sequence of words (and intervening or preceding pauses) drove classification.

## Results

### Demographics & spectrograms

The schizophrenia-spectrum cohort included individuals spanning multiple points along the illness trajectory, from clinically high-risk and first-episode psychosis to long-standing schizophrenia and schizoaffective disorders (Table 1). This broad range of diagnostic subtypes contributes to the ecological validity of our sample by capturing a variety of symptom intensities and illness stages. Although there was no significant difference in age compared to controls, men were overrepresented among patients—consistent with known epidemiologic patterns [50]. No sex or age differences were present in the patient median or blunted affect groups. Medication exposure among SSD participants varied considerably, ranging from unmedicated clinical high-risk individuals to first-episode psychosis patients with less than three days of exposure to antipsychotic use [34], and extending to longer-term cases receiving long-acting injectable medications. While we did not control for individual dosage and medication, the group proportions of type of medication usage were matched across train, validation, and test splits. As such we cannot equate the observed acoustic classification performance to primary vs secondary (medication-related) negative symptoms.

The split on the PANSS-8 negative symptom N1+N4+N6 items average was performed for a median score of 7 (minimum 3; maximum 21) resulting in 97 participants below and 82 participants above the median. For the blunted affect split, 119 SSD participants had an N1 score 3 or below, with 60 participants having a score of 4 or higher. Table 2a, b provide further demographic and clinical details for the negative-symptom and blunted-affect sub cohorts, respectively.

**Table 2:** Demographic and clinical characteristics of clinical sub-cohorts.

a				
Demographics of SSD patients with sub-median average negative PANSS scores against SSD patients with above-median average negative PANSS scores.				
Category		Sub-median average negative PANSS score (*n* = 97)	Above-median average negative PANSS score (*n* = 82)	Statistics
Age				
Years	M (SD)	27.4 (7.04)	28.27 (11.51)	*F* = *0.379, p* = *0.539*
Sex				
Male	*n (%)*	70 (72.16)	66 (80.49)	X^2^ = 1.2613, *p* = 0.2614
Clinical scores				
N1	M (SD)	1.39 (0.67)	4.23 (1.28)	*F* = *361, p* < *0.001*^a^
N1, N4, N6	M (SD)	4.26 (1.56)	12.11 (3.45)	*F* = *404.6, p* < *0.001*^a^

b				
Demographics of SSD patients with a N1 score ≤3 against SSD patients with a N1 score >3				
Category		Mildly ill patients (PANSS N1 score ≤ 3, *n* = 119)	Severely ill patients (PANSS N1 score > 3, *n* = 60)	Statistic
Age				
Years	M (SD)	26.98 (7.18)	29.38 (12.44)	*F* = *2.632, p* = 0.107
Sex				
Male	*n* (%)	88 (73.95)	48 (80)	X² = 0.50288, *p* = 0.4782
Clinical scores				
N1	M (SD)	1.62 (0.81)	4.82 (0.91)	*F* = *567.6, p* < 0.001^a^
N1, N4, N6	M (SD)	5.18 (2.46)	13.15 (3.43)	*F* = *318.2, p* < 0.001^a^

*M* mean, *SD* standard deviation, *PANSS* positive and negative syndrome scale, *NOS* not otherwise specified, *FEP* first episode psychosis, *CHR* clinical high risk.

^a^Indicates *p* value < 0.001.

Duration of participant-only audio recordings for the control group were on average 517 s long, with a minimum duration of 108 and a maximum of 1972 s (SD - 331 s) while for SSD cohort the average was 410 s (min - 51 s, max 1594, SD 316 s). Note that these durations are the total duration only of the extracted, uninterrupted 10 s fragments per interview which were assigned to the participant. The spectrogram pipeline resulted in an SSD patients & HCs cohort of 110,246 unique spectrograms. The negative symptom cohorts containing only SSD patients had 59,562 unique spectrograms each.

### Classifiers

Classification results for the three tasks on a per-spectrogram basis are summarized in Table 3. Overall, the *diagnostic classifier* showed strong performance, attaining an AUC of 0.8651 and yielding 87.83% test accuracy. Within this framework, healthy controls were identified with a moderate precision (0.7446) and high recall (0.8365), whereas the schizophrenia-spectrum group displayed notably higher precision (0.9366) and a robust F1 score of 0.9147.

**Table 3:** Performance metrics of classifiers.

	AUC	Test accuracy (%)	Precision	Recall	F1 score
Diagnostic classifier					
HC	0.8651	87.83	0.7446	0.8365	0.7879
SSD			0.9366	0.8938	0.9147
Median-split classifier					
Sub-median	0.733	80.46	0.9627	0.8188	0.8849
Above-median			0.2431	0.6473	0.3535
Blunted affect (N1) classifier					
Mildly ill (N1 ≤ 3)	0.7856	87.84	0.9734	0.8936	0.9318
Severely ill (N1 > 3)			0.325	0.6776	0.4393

For the *negative symptom severity classifier*, designed to distinguish more severely ill from less severely ill patients based on overall symptom severity, accuracy was 80.46% though the AUC dropped to 0.7330. As the precision and F1 scores indicate, the sub-median (“less severe”) group was identified with substantially higher certainty than the above-median (“more severe”) group. Finally, *the blunted affect (N1) classifier* achieved 87.84% accuracy, along with an AUC of 0.7856. Though the mildly ill subgroup (N1 ≤ 3) was classified with near-perfect precision (0.9734) and high recall, 0.893, the more severely ill subgroup’s performance was notably lower, mirroring the pattern seen in the median-split task.

When we applied the blunted affect classifier to the negative severity-based classification task, performance declined notably, with the AUC dropping to 0.5831. Similarly, the negative severity-based classification classifier applied to the blunted affect dropped to an AUC of 0.5334. Note that the blunted affect and severity splits were trained on the same spectrograms of the same SSD participants, but divided differently. This decline in performance indicates that the blunted affect models are indeed capturing symptom-specific acoustic patterns rather than general illness severity and vice versa, as indicated by the contrast with their stronger original results on the negative-symptom classification task. Diagnostic classifier performance on negative symptom severity ratings dropped to an AUC of 0.5046 with an accuracy of 0.6482, while for the N1 division it dropped to 0.4954 (test-set accuracy of 0.3518). See supplemental Table S1 for all metrics of trained model performance on different divisions.

When averaging spectrograms on a subject basis to achieve per-participant scores, accuracy was similar for diagnostic (87.8% spectrogram-level; 89.8% participant-level), dropped somewhat for median symptom classification (80.5% vs 71.4%) and was similar for N1 detection (87.84% vs 85.7%).

Comparing the CNN approach to the wav2vec2 and eGeMAPSv02 methods showed CNN accuracy outperforming them on diagnosis (87.83, 81.6 and 71.4% for CNN,wav2vec and eGeMAPSv02, respectively), median split (81.6, 75.0 and 71.4% respectively) and N1 (87.8, 71.4 and 75.0% respectively) - but eGeMAPSv02 had a higher AUC for N1 classification (0.856 versus 0.786 for CNN; for full results see Supplementary Table S2).

### Grad-CAM visualization results

Grad-CAM heatmaps revealed a progressive refinement of activations across the five convolutional blocks [(7 × 7, 64), (3 × 3, 64), (3 × 3, 128), (3 × 3, 256) and (3 × 3, 512)], with each stage focusing more sharply on spectro-temporal features of interest (Fig. 1). In the final layer, these regions aligned with stretches of speech in the mid-frequency range, suggesting the model was leveraging information tied to vocal articulation and resonance.

Next, examining the longest contiguous activation above 63% of maximum intensity allowed us to gauge how much continuous speech shaped the network’s decisions. In the SSD-versus-HC classifier, the median uninterrupted activation was 5.45 s for controls and 6.61 s for patients, indicating that entire phrases rather than isolated phonemes were implicated. Similarly, for blunted affect detection, median active durations were 6.52 s (N1/123) and 5.27 s (N1/4567), suggesting that multiple consecutive words, along with their intervening pauses, played a key role in classification.

## Discussion

Our findings indicate that analyzing spectrograms of brief, 10 s speech segments can reliably identify a range of psychotic disorders that occur in schizophrenia spectrum and the individuals with clinically notable blunted affect, a core negative symptom, with sufficient accuracy (AUC > 0.75). By preserving both acoustic details (pitch, prosody) and short-term temporal cues (pauses, shifts in vocal energy), our CNN-based approach highlights the importance of moment-to-moment fluctuations in speech—features often lost when data are averaged across entire recordings. From a theoretical standpoint, this work underscores the role of negative symptoms in modulating subtle aspects of speech production [51, 52]. Moreover, by specifically isolating blunted affect as one symptom of interest, we demonstrate that a single clinical construct can be captured from short vocal samples. Data-driven models proved capable of extracting these cues without relying on handcrafted metrics, suggesting that speech-based markers of blunted affect or overall symptom severity can be detected in short conversational fragments.

Our results confirm previous findings suggesting that acoustic speech features can serve as reliable biomarkers for the distinguishment of schizophrenia spectrum disorder patients from healthy controls [9, 53]. We further confirm previous findings that acoustic speech features may serve not only as reliable biomarkers for distinguishing schizophrenia spectrum disorder patients from healthy controls, but also in classifying the severity of the disorder [9, 54].

Our diagnostic classifier (AUC = 0.865) is more accurate than our symptom severity classifiers (AUC = 0.733, AUC = 0.786). This can be explained by the presence of at least some symptoms in both patient groups, making the differences between groups smaller and thus making classification harder. Identifying individuals with prominent blunted affect (N1) was more accurate (AUC = 0.7856) than identifying those with higher overall negative symptom burden (AUC = 0.7330). This is to be expected as blunted affect (N1) is a primarily acoustic feature, while the other negative symptoms (N4 and N6) require an assessment of pragmatic aspects of one’s interaction with others [39].

Our diagnostic classifier did not perform better than chance when applied to identify symptom severity (AUCs <0.51). Of note, acoustic features that correlate with negative symptoms are known to contribute to the diagnostic separation of schizophrenia [55], but to our knowledge cross-label applications of severity and diagnostic markets have not been reported to date. Our results indicate that the pattern contributing to acoustic separation of diagnostic category is not the same as the pattern that marks symptom severity. This resonates with the fact that clinicians often use distinct features to identify diagnoses (i.e., presence or absence of a set of defined symptoms), but employ a different approach to appraise the severity of a patient’s condition (frequency, distress and functional effects of those symptoms).

We obtained a lower AUC in our diagnostic classifier than some previous literature using a similar approach (AUC = 0.9978, [24]). However, it is important to note that we had access to a larger, more heterogeneous sample of 227 patients, while Fu et al. recruited 56 patients for a single study. Existing work has often used narrowly defined cohorts, limiting how well findings generalize across the wide range of illness stages and symptom presentations that characterize the schizophrenia spectrum [8, 24, 55–57].

Concurrent with a heterogeneous data set, preventing data leakage is vital in clinical machine learning, as inadvertently reusing the same participant’s data in both training and testing can lead to overly optimistic performance estimates and hamper real-world applicability [58–61]. In many biomedical studies, large datasets are segmented at the slice, patch, or short-segment level under cross-validation, inadvertently allowing identical or near-identical information to appear across folds. To avoid this pitfall, we enforced a strict participant-level split for training, validation, and test sets, while also balancing the proportion of samples sourced from each original study. This approach preserves independence between datasets, better reflects actual clinical usage scenarios, and provides a robust measure of the model’s true predictive power.

### Strengths and limitations

Our approach features a large, well-defined multi-stage sample capturing the real-world clinical population to whom the eventual results will be applicable. Our strict partitioning of training, validation, and test sets, ensured a realistic estimate of model performance, with transparent insights into the model’s decision making based on Grad-CAM analysis. Compared to alternative algorithms, such as wav2vec, that learn representations from raw waveforms without spectrograms [26], Grad-CAM provided the visual interpretability required to precisely pinpoint the time–frequency plane critical for the classificatory performance. Despite the strengths of our transparent, clinically interpretable outputs, we have a few limitations that require further consideration. We did not control for dosage of medication on a participant level, although we balanced type (i.e. medication-naive, long-acting injectable) across test, train and validation sets. We restricted our analysis to a single language - English, which may limit generalizability to other languages [14]. In addition, we concentrated on only detecting one specific symptom—blunted affect—while the symptoms in schizophrenia encompass a broader range of phenomena (e.g., social withdrawal, poverty of speech). Future studies could expand on these points by examining multilingual datasets and addressing a more diverse array of symptom constructs.

Diagnostic classification had a strong performance, achieving an accuracy of 87.8%1. Within the patients, performance was lower (80.46% for negative symptoms) or similar (87.84% for blunted affect). Because this margin is small, and because the split group size for the symptom divisions decreases the opportunity for the model to adequately train; results for split groups should be interpreted with more caution than our patient/control division. We kept a single, stratified train/validation/test split mirroring clinical deployment while aiming to preserve balanced heterogeneity of diagnosis, illness stage, type of medication and sex across partitions. The objective was not to chase peak accuracy but to show that a raw spectrogram CNN can classify disorder and pick up specific symptom profiles. Looking ahead, mapping which frequency–time domains drive classifications across larger samples and sites, or with careful application of data augmentation preserving set independence [47], may yield more granular and robust symptom detectors.

Our findings underscore the promise of a CNN-based analysis of short speech segments for both diagnosing schizophrenia spectrum disorders and gauging negative-symptom severity. By retaining temporal structure and acoustic detail, this method captures important nuances often lost in simpler feature-averaging strategies. With further refinements—such as multilingual datasets or expanded symptom domains—these approaches could become increasingly relevant for clinical assessment and longitudinal symptom tracking.

#### Citation diversity statement

The authors have attested that they made efforts to be mindful of diversity in selecting the citations used in this article.

## Supplementary information

Supplemental Table 1

Supplemental Table 2

## Data Availability

For DISCOURSE-UWO and TOPSY, anonymised data are made available to qualified researchers through https://talkbank.org/psychosis/, a collaberation between the DISCOURSE in Psychosis consortium (https://discourseinpsychosis.org/) and TalkBank. Restrictions apply and conditions are accessible via the TalkBank URL above. For IMPLEMENT, raw audio fragments and spectrograms are privacy-sensitive personal data and cannot be shared. For anonymous eGeMAPSV02 patient-level features, the corresponding author can be contacted.
