# Supplementary material for: Taking a look at your speech: identifying diagnostic status and negative symptoms of psychosis using convolutional neural networks
Source: NPP Digit Psychiatry Neurosci. 2025 Jul 8;3:19. doi: 10.1038/s44277-025-00040-1 (PMC12237691; doi:10.1038/s44277-025-00040-1)
Supplement: Supplementary file 1 — Supplemental Table 1 [file 44277_2025_40_MOESM1_ESM.docx]

| **Supplementary Table 1** | |  |  |  |  |  |  |
| --- | --- | --- | --- | --- | --- | --- | --- |
| Performance metrics of classifiers on other datasplits. | | |  |  |  |  |  |
|  |  |  | AUC | Test accuracy (%) | Precision | Recall | F1 score |
| **Diagnostic classifier** | |  |  |  |  |  |  |
|  | **Median-split division** | Sub-median | 0.5046 | 0.6482 | 0.6887 | 0.8896 | 0.7764 |
|  |  | Above-median |  |  | 0.3308 | 0.1195 | 0.1756 |
|  |  |  |  |  |  |  |  |
|  | **Blunted affect division** | Mildly ill (N1 ≤ 3) | 0.4954 | 0.3518 | 0.6692 | 0.1104 | 0.1896 |
|  |  | Severely ill (N1 > 3) |  |  | 0.3113 | 0.8805 | 0.4600 |
|  |  |  |  |  |  |  |  |
| **Negative symptoms median-split classifier** | | |  |  |  |  |  |
|  | **Blunted affect division** | Mildly ill (N1 ≤ 3) | 0.5334 | 0.3219 | 0.8875 | 0.2298 | 0.3651 |
|  |  | Severely ill (N1 > 3) |  |  | 0.1627 | 0.837 | 0.2724 |
|  |  |  |  |  |  |  |  |
| **Blunted affect (N1) classifier** | |  |  |  |  |  |  |
|  | **Median-split division** | Sub-median | 0.5831 | 0.7423 | 0.7919 | 0.894 | 0.8399 |
|  |  | Above-median |  |  | 0.4533 | 0.2721 | 0.3401 |
| Note: Test Results of the Median split and N1 classifiers on diagnostic data are unavailable, since by definition all the patients in the dataset of the median and N1 split classifier are in the PT category. | | | | | | | |
| Legend: AUC = area under the curve | | | | | | | |
