# Supplementary material for: Taking a look at your speech: identifying diagnostic status and negative symptoms of psychosis using convolutional neural networks
Source: NPP Digit Psychiatry Neurosci. 2025 Jul 8;3:19. doi: 10.1038/s44277-025-00040-1 (PMC12237691; doi:10.1038/s44277-025-00040-1)
Supplement: Supplementary file 2 — Supplemental Table 2 [file 44277_2025_40_MOESM2_ESM.docx]

| **Supplementary Table 2** | |  |  |
| --- | --- | --- | --- |
| Performance metrics of various classification modalities. | | |  |
|  |  | AUC | Test accuracy (%) |
| **CNN (ResNet18)** |  |  |  |
|  | **Diagnostic classifier** | 0.8651 | 87.8 |
|  |  |  |  |
|  | **Median-split classifier** | 0.7330 | 80.5 |
|  |  |  |  |
|  | **Blunted affect (N1) classifier** | 0.7856 | 87.8 |
|  |  |  |  |
| **Wav2Vec2** |  |  |  |
|  | **Diagnostic classifier** | 0.696 | 81.6 |
|  |  |  |  |
|  | **Median-split classifier** | 0.538 | 75.0 |
|  |  |  |  |
|  | **Blunted affect (N1) classifier** | 0.296 | 21.4 |
|  |  |  |  |
| **eGeMAPSv02** |  |  |  |
|  | **Diagnostic classifier** | 0.721 | 71.4 |
|  |  |  |  |
|  | **Median-split classifier** | 0.713 | 71.4 |
|  |  |  |  |
|  | **Blunted affect (N1) classifier** | 0.856 | 75.0 |

**Table S2** - Performance metrics of CNN approach compared to Wav2vec2 and openSMILE eGemapsv02. For both alternative approaches, audio fragments were concatenated per participant. The Wav2vec2 approach used the wav2vec2-base-960h model trained on the training & validation set, with a binary final layer for classification. We resampled participant audio to 16 kHz and passed it through the wav2vec 2.0 base model; the feature encoder was frozen, and a new classification head fine-tuned for five epochs (learning rate 1 × 10-5, 5 epochs). The openSMILE approach used the eGeMAPSv02 88-feature set, with a random forest classifier (600 trees using balanced class weights); testing data performance is reported.
